# Point-of-Care Ultrasound Image of Intra-Abdominal Lymphadenopathy in Tuberculosis

**DOI:** 10.4269/ajtmh.20-0697

**Published:** 2020-12

**Authors:** Chalese O. Einbeck, Christian John Hunter, Kosuke Yasukawa

**Affiliations:** 1 School of Medicine, Faculty of Health Sciences, University of Namibia, Windhoek, Namibia;; 2 Division of Hospital Medicine, Department of Medicine, MedStar Washington Hospital Center, Washington, District of Columbia

A previously healthy 24-year-old woman presented to the internal medicine department with a 4-month history of frequent loose stools, abdominal pain, unintentional weight loss, and fever. On physical examination, she was severely cachectic with marked temporal wasting; there was no significant cervical, axillary, or inguinal lymphadenopathy. The abdomen was scaphoid but otherwise unremarkable. A routine stool culture was negative for bacterial pathogens, and stool examination for ova and parasites was negative. The result for the *Clostridioides difficile* stool test was negative. The HIV antibody test was negative. Chest radiograph showed minimal left upper zone opacification. Three successive sputum samples were submitted for *Mycobacterium tuberculosis* PCR, and the results were negative. Tuberculosis urine lipoarabinomannan and PCR were also negative. Point-of-care abdominal ultrasound revealed significant intra-abdominal lymphadenopathy, some of which contained central areas of calcification and ascites in the abdomen. Figure 1 shows both the unlabeled (on left) and labeled abdominal frame capture with large lymph nodes with hyperechoic central enhancement. Supplemental Videos 1 and 2 reveal further fluid, bowel loops, and several large lymph nodes. Biopsy of the lymph node could not be pursued because of lack of interventional radiology services and the risk of open biopsy. The patient was empirically started on rifampicin, isoniazid, pyrazinamide, and ethambutol. Over the course of her hospital stay, a repeat tracheal aspirate eventually yielded a positive PCR/Xpert result for *M. tuberculosis*. There was marked clinical improvement after the first week of antituberculosis treatment. The patient was discharged home 3 weeks after this treatment was initiated.

**Figure 1.**
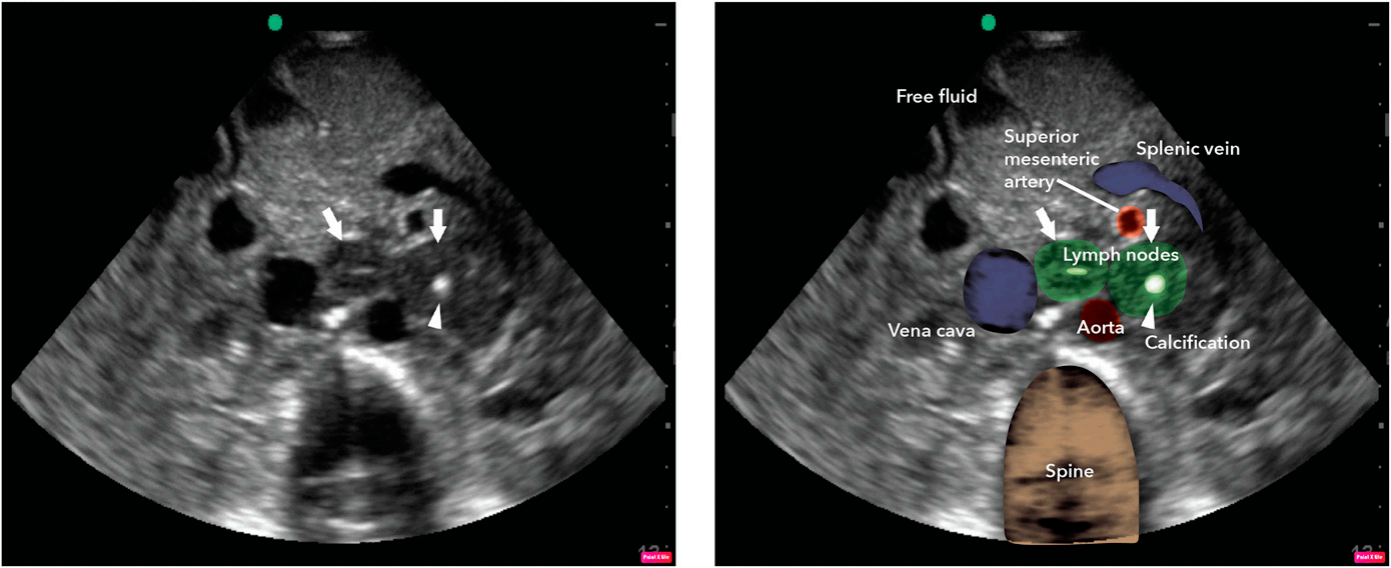
Unlabeled (left) and labeled abdominal frame capture with large lymph nodes with hyperechoic central enhancement. This figure appears in color at www.ajtmh.org.

Intra-abdominal lymphadenopathy is a common manifestation of extrapulmonary tuberculosis and disseminated tuberculosis. Intra-abdominal lymphadenopathy due to tuberculosis appears as hypoechoic round structures which are considered pathological when larger than 1.5–2 cm.^1^ The lymph nodes may have a more hypoechoic center due to caseous necrosis, and in the late stage, calcifications may be seen.^2^ Evaluation of intra-abdominal lymphadenopathy is part of the sonographic protocol for assessment of tuberculosis in patients with HIV.^3^ Point-of-care ultrasound is a useful tool in resource-limited settings in the detection of intra-abdominal lymphadenopathy.

## Supplemental videos

Supplemental materials
