# Supplementary material for: Point-of-Care Ultrasound Image of Intra-Abdominal Lymphadenopathy in Tuberculosis
Source: Am J Trop Med Hyg. 2020 Dec;103(6):2156–7. doi: 10.4269/ajtmh.20-0697 (PMC7695061; doi:10.4269/ajtmh.20-0697)
Supplement: Supplementary file 1 [file tpmd200697.SD1.docx]

Suppl Video_1. TB_lymphadenopathy.

Suppl Video_2. TB_intraabdominal_lymphadenopathy_2.

*Note*: Supplemental Videos 1 and 2 will be available online in final publication.

\
